# Calcium‐sensing receptor induces the apoptosis of chondrocytes in cooperation with phosphate transporter

**DOI:** 10.1002/2211-5463.70142

**Published:** 2025-10-13

**Authors:** Sachie Nakatani, Hiroya Ueda, Ayumi Kawata, Yuki Sato, Kotomi Inomata, Hiroshi Mano, Masahiro Wada, Kenji Kobata

**Affiliations:** ^1^ Department of Pharmaceutical Sciences, Faculty of Pharmacy and Pharmaceutical Sciences Josai University Sakado Saitama Japan; ^2^ Department of Clinical Dietetics and Human Nutrition, Faculty of Pharmacy and Pharmaceutical Sciences Josai University Sakado Saitama Japan

**Keywords:** apoptosis, calcium‐sensing receptor, chondrocyte, mineralization, phosphate transporter

## Abstract

Excess extracellular inorganic phosphate ions (Pi) and calcium ions (Ca^2+^) cause apoptosis and subsequent mineralization of chondrocytes. Here, we investigated the mechanism underlying the effect of these minerals. The chondrogenic cell line ATDC5 was treated with 2 mm Pi and/or Ca^2+^, and apoptosis, mineralization, and intracellular Pi concentrations were determined. Further, Pi‐ and Ca^2+^‐treated cells were incubated with the Pi transporter (Pit‐1) blocker phosphonoformic acid (PFA), the calcium‐sensing receptor (CaSR) antagonist NPS‐2143, and the CaSR agonist GdCl_3_. Individual addition of Pi and Ca^2+^ did not induce apoptosis and mineralization, while combined addition of the minerals induced both. The Pit‐1 blocker and the CaSR antagonist completely inhibited the apoptosis induced by combined addition of Pi and Ca^2+^. Intracellular Pi concentration was remarkably increased by combined addition of Pi and Ca^2+^ as compared to the findings for individual addition. The Pit‐1 blocker and CaSR antagonist completely inhibited the increase in intracellular Pi concentration induced by the combined addition of Pi and Ca^2+^. The CaSR agonist considerably increased the intracellular Pi concentration. Our results indicate that excess extracellular Ca^2+^ activates CaSR, which induces the intake of excess extracellular Pi through Pit‐1 into ATDC5 cells. The resulting increase in intracellular Pi concentration induces apoptosis.

## Graphical abstract

Excess Ca^2+^ ions activate the Calcium‐Sensing Receptor (CaSR), which subsequently drives the uptake of excess inorganic phosphate (Pi) via the Pi transporter (Pit−1) in chondrocytes. This mechanism causes a toxic increase in intracellular Pi concentration, ultimately leading to chondrocyte apoptosis and pathological mineralization.

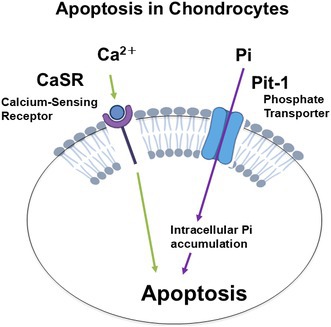

**Abbreviations**
ALP: alkaline phosphatase
Ca^2+^
: calcium ions
CaSR: calcium‐sensing receptor
DMEM/F12: 1 : 1 mixture of Dulbecco's Modified Eagle's and Ham's F12 Medium
EDTA: ethylenediaminetetraacetic acid
GdCl_3_
: Gadolinium (III) chloride hexahydrate
PBS (−): Ca^2+^‐ and Mg^2+^‐free phosphate‐buffered saline
PFA: Phosphonoformic acid
Pi: phosphate ions
Pit‐1: phosphate transporter

Growth of the long bones is accomplished by chondrocyte mineralization in the growth plate cartilage through chondral differentiation. During this process, the chondrocytes proliferate, mature, hypertrophy, and differentiate into calcified chondrocytes; finally, apoptosis is triggered by an increase in the extracellular mineral concentration [1, 2]. Following apoptosis, the cartilage is invaded by blood vessels, osteoclasts, and osteoblasts, with the latter two cell types facilitating the replacement of cartilage with bone [3].

Mansfield *et al*. reported that the combination of excess extracellular inorganic phosphate ions (Pi) and excess calcium ions (Ca^2+^) induced chondrocyte apoptosis via a phosphate transporter, Pit‐1 [4]. They reported that treatment with a general Ca^2+^ channel inhibitor also did not protect from excess Pi‐ and Ca^2+^‐induced apoptosis [5]. Therefore, the detailed mechanism of Ca^2+^‐inducing apoptosis is unclear. Other studies showed that chondrocytes possessed an extracellular Ca^2+^ sensing mechanism called calcium‐sensing receptor (CaSR), and it modulated chondrocyte mineralization [6, 7, 8]. Our previous *in vitro* study showed that excess extracellular Ca^2+^ induced the calcification of chondrocytes via CaSR [9]. However, how CaSR is involved in the apoptosis process induced by Ca^2+^ and Pi is unknown. Recent work has highlighted that CaSR signaling interacts with phosphate metabolism and affects mineralization and apoptosis in multiple tissues, including bone and cartilage [10, 11, 12, 13]. In this study, we investigated how co‐incubation of excess extracellular Pi‐ and Ca^2+^‐induced apoptosis and subsequent mineralization in the chondrogenic cell line ATDC5 and how CaSR is involved in the events.

## Materials and methods

### Cell culture

A mouse embryo chondrogenic cell line, ATDC5, was purchased from RIKEN Cell Bank (Ibaraki, Japan). The cells were cultured in a 1 : 1 mixture of Dulbecco's Modified Eagle's and Ham's F12 Medium (DMEM/F12; Life Technologies Japan Ltd., Tokyo, Japan) supplemented with 5% fetal bovine serum (Nichirei Biosciences Inc., Tokyo, Japan) and penicillin (50 U·mL^−1^)–streptomycin (50 mg·mL^−1^) (Meiji Seika Pharma Co. Ltd., Tokyo, Japan). The culture was maintained at 37 °C in a humidified atmosphere of 5% CO_2_/95% air.

### Treatment reagents

The initial concentration of Pi and Ca^2+^ in the serum‐free control medium was 1.0 mm for both. The final concentration of 2 mm Pi and 2 mm Ca^2+^ was prepared by adding NaH_2_PO_4_·2H_2_O (Wako Pure Chemical Inc. Ltd., Osaka, Japan) and CaCl_2_·2H_2_O (Sigma‐Aldrich, St. Louis, MO, USA) in the control medium. Phosphonoformic acid (PFA) was purchased from Santa Cruz Biotechnology, Inc. (Dallas, TX, USA). NPS‐2143 was purchased from MedChem Express Co. Ltd. (Princeton, NJ, USA). Gadolinium (III) chloride hexahydrate (GdCl_3_) was purchased from Sigma‐Aldrich.

### Measurement of cell viability

ATDC5 cells were seeded at 2.0 × 10^3^ cells/0.32‐cm^2^ well. After 24‐h incubation, the cells were further incubated with medium containing a combination of 2 mm Pi, 2 mm Ca^2+^, 0.5 mm PFA, 0.05 μm NPS‐2143, and 10 μm GdCl_3_ for 3 days. PFA was readily soluble in culture medium and was therefore directly dissolved in the medium at the desired concentration. NPS‐2143 was first dissolved in dimethyl sulfoxide (DMSO) to prepare a 1 mm stock solution and then diluted with culture medium to a final concentration of 0.05 μm. This medium was replaced by a medium containing 10% WST‐1 reagent (Roche Co. Ltd., Basel, Switzerland), and the cells were then incubated for 3 h at 37 °C. The absorbance at 440 nm was measured with a microplate reader (SpectraMax M2e; Bio‐Rad, Hercules, CA, USA).

### Measurement of cell apoptosis

ATDC5 cells were seeded at 2.0 × 10^4^ cells/1.88‐cm^2^ well. After 24‐h incubation, the cells were further incubated with medium containing 2 mm Pi and/or 2 mm Ca^2+^ for 3 days. The cells were rinsed with minus Ca^2+^‐ and Mg^2+^‐free phosphate‐buffered saline (PBS (−)) and collected using trypsin/ethylenediaminetetraacetic acid (EDTA; Sigma‐Aldrich). Apoptotic cells were measured with the Annexin V and Dead Cell kit (Merck Co., Tokyo, Japan) using MUSE Cell Analyzer (Merck).

### Measurement of cell mineralization

ATDC5 cells were seeded at 2.0 × 10^4^ cells/1.88‐cm^2^ well. After 24‐h incubation, the cells were further incubated with medium containing 2 mm Pi and/or 2 mm Ca^2+^ for 10 days. The cells were then fixed in 10% formalin (Wako), and the calcium deposits were stained with 0.5 g·mL^−1^ Alizarin red reagent (pH 6.3; Sigma‐Aldrich) dissolved in deionized water. The stained area was analyzed using ImageJ ver 1.6 (NIH, Bethesda, MD, USA).

### Measurement of alkaline phosphatase (ALP) activity

ATDC5 cells were seeded at 2.0 × 10^4^ cells/1.88‐cm^2^ well. After 24‐h incubation, the cells were further incubated with medium containing 2 mm Pi and/or 2 mm Ca^2+^ for 3 days. The cells were fixed with 10% formalin, and ALP activity was determined by treatment with 2.5 mg·mL^−1^ naphthol (Sigma‐Aldrich) and 2.5 mg·mL^−1^ Fast Red (Sigma‐Aldrich) dissolved in 0.44 g·mL^−1^ 2‐amino‐2‐methyl‐1‐propanol solution. The stained areas were analyzed using Image J.

### Measurement of intracellular pi concentration

ATDC5 cells were seeded at 2.0 × 10^5^ cells/20.8‐cm2 well. After 24‐h incubation, the cells were further incubated with medium containing a combination of 2 mm Pi, 2 mm Ca^2+^, 0.5 mm PFA, 0.05 μm NPS‐2143, and 10 μm GdCl_3_ for 3 days. The cells were rinsed with PBS (−) and collected using trypsin/EDTA. The number of cells was adjusted to 5.0 × 10^5^ cells·mL^−1^. After the cells were washed three times with a buffer containing 135 mmol·L^−1^ NaCl, 100 mmol·L^−1^ mannitol (Wako), 10 mmol·L^−1^ HEPES (Dojindo Laboratories Inc., Kumamoto, Japan), and 1 mmol·L^−1^ PFA (pH 7.5), the intracellular Pi was extracted from the cells with 250 μL ice‐cold 3% perchloric acid (Wako). After centrifugation at 16 000 ***g*** for 20 min, the Pi concentration of the supernatant obtained was measured with the Malachite Green Phosphate Assay Kit (BioAssay Systems, Hayward, CA, USA). The absorbance at 620 nm was measured with a microplate reader.

### Statistical analyses

Data have been expressed as the mean ± SD. The number of measurements is shown in the figures. Statistical analyses were carried out using the JMP software (JMP Statistical Discovery LLC., Tokyo, Japan). Statistical analysis was performed using one‐way analysis of variance (ANOVA). When a significant difference was observed (*P* < 0.05), post hoc pairwise comparisons were conducted using Welch's *t*‐test with Bonferroni correction to adjust for multiple comparisons.

## Results

### Effects of Pi and Ca^2+^ on viability and apoptosis rate of ATDC5 cells

**Fig. 1:**
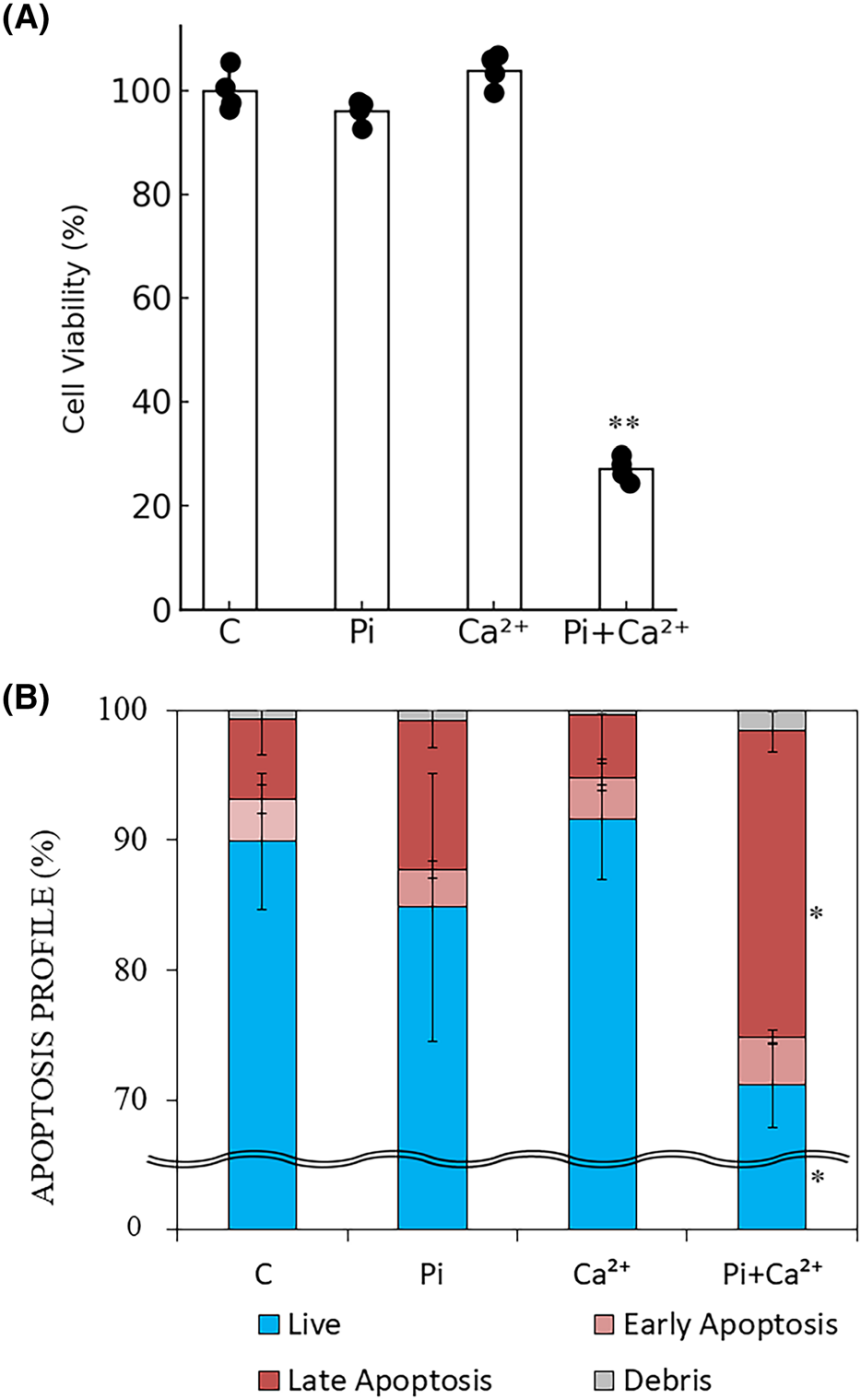
Effects of Pi and Ca^2+^ on ATDC5 cells. ATDC5 cells were cultured in differentiation medium and treated with 2 mm inorganic phosphate (Pi) and/or 2 mm calcium (Ca^2+^) for 72 h. (A) Cell viability was assessed using the WST‐1 assay and expressed as the percentage relative to untreated control cells. (B) Apoptosis profiles were determined using the Annexin V & Dead Cell Kit followed by flow cytometry. Blue areas represent live cells, pink areas indicate early apoptotic cells, red areas represent late apoptotic cells, and gray areas indicate debris. Statistical analysis was performed for each cell stage in comparison with the control group. Data are presented as mean ± SD (*n* = 4 wells per group), and the experiments were repeated three times with similar results. Statistical significance was determined using one‐way ANOVA, followed by Welch's *t*‐test for *post hoc* pairwise comparisons (*P < 0.05, **P < 0.01 vs. C).

First, we attempted to reconfirm the event that the apoptosis of ATDC5 cells occurs only when both excess Pi and Ca^2+^ are present outside the cells. Individual addition of either Pi or Ca^2+^ did not affect cell viability, but the combined addition of the two significantly reduced cell viability (Fig. 1A). The populations of late‐stage apoptotic ATDC5 cells were significantly increased by the combined addition of Pi and Ca^2+^ (Fig. 1B).

### Effects of Pi and Ca^2+^ on viability and apoptosis rate of ATDC5 cells

**Fig. 2:**
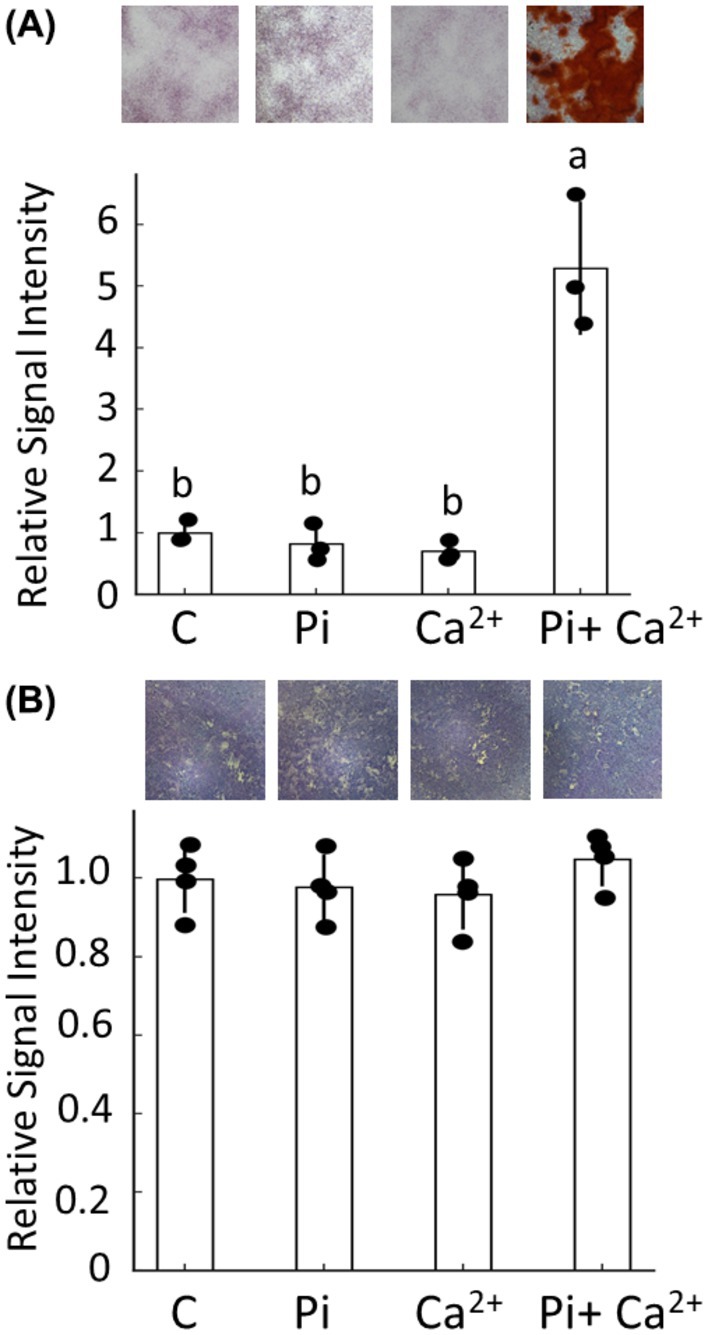
Effects of Pi and Ca^2+^ on mineralization and ALP activity in ATDC5 cells. (A) Calcium deposition was evaluated after 10 days by Alizarin red staining, with representative dish images shown as insets illustrating calcium deposition under each condition. The relative staining area was quantified using the ImageJ software and normalized to the control (set as 1.0); values > 1 indicate increased mineralization. Data are shown as mean ± SD (*n* = 3 wells per group). Statistical significance was determined using one‐way ANOVA followed by Welch's *t*‐test with Bonferroni correction. Bars labeled with different superscript letters (a, b, c) are significantly different from each other at *P* < 0.05, whereas groups sharing the same letter are not significantly different. Superscript letters represent outcomes of *post hoc* multiple comparisons and do not correspond to a specific reference group. (B) ALP activity was assessed after 3 days using naphthol/Fast Red staining. Stained areas were quantified using ImageJ. Data are shown as mean ± SD (*n* = 4 wells per group), and experiments were repeated three times with similar results. Statistical analysis was performed as described above, and significant group differences are indicated by distinct superscript letters.

Next, we examined whether excess extracellular Pi and Ca^2+^ affect the differentiation and mineralization of ATDC5. Calcification and differentiation of ATDC5 cells were evaluated by staining the calcium deposits and measuring the activity of ALP, respectively. Individual addition of Pi and Ca^2+^ did not affect the mineralization, but combined addition remarkably increased the mineralization (Fig. 2A). No alterations were noted in ALP activity under any conditions (Fig. 2B).

### Effect of Pit‐1 blocker and CaSR antagonist on cell viability

**Fig. 3:**
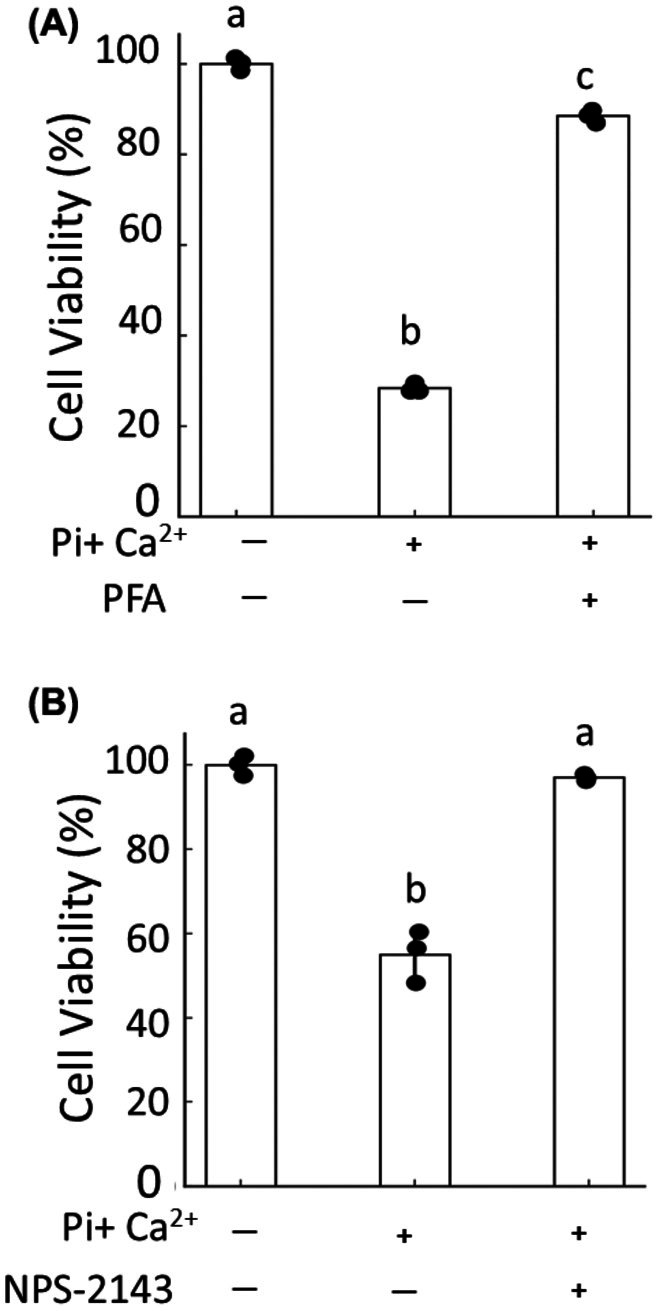
Effects of PFA and NPS‐2143 on Pi + Ca^2+^‐induced apoptosis of ATDC5 cells. ATDC5 cells were treated for 3 days with 2 mm Pi and 2 mm Ca^2+^ in the presence or absence of either 0.5 mm phosphonoformic acid (PFA, a Pit1 blocker) or 0.05 μm NPS2143 (a CaSR antagonist). Cell viability was subsequently assessed using the WST1 assay. (A) Effect of PFA on Pi+‐Ca^2+^induced apoptosis. (B) Effect of NPS2143 on Pi+Ca^2+^‐induced apoptosis. Data are shown as mean ± SD (*n* = 3 wells per group), and experiments were repeated three times with similar results. Statistical significance was determined using one‐way ANOVA followed by Welch's t‐test with Bonferroni correction. Different superscript letters (a, b, c) denote statistically distinct groups at *P* < 0.05; the same letter indicates no significant difference.

Therefore, we investigated whether the apoptosis induced by the combined addition of Pi and Ca^2+^ was mediated through Pit‐1 and/or CaSR. PFA, a blocker of Pit‐1 [14, 15, 16] completely inhibited the decrease in cell viability induced by the combined addition of Pi + Ca^2+^ (Fig. 3A). Moreover, NPS‐2143, a CaSR antagonist [17], also completely inhibited the decrease in cell viability (Fig. 3B). As a result, apoptosis of chondrocytes might be caused by the cooperative action of CaSR in addition to Pit‐1.

### Effects of Pi and Ca^2+^ on intracellular Pi concentration of ATDC5 cells

**Fig. 4:**
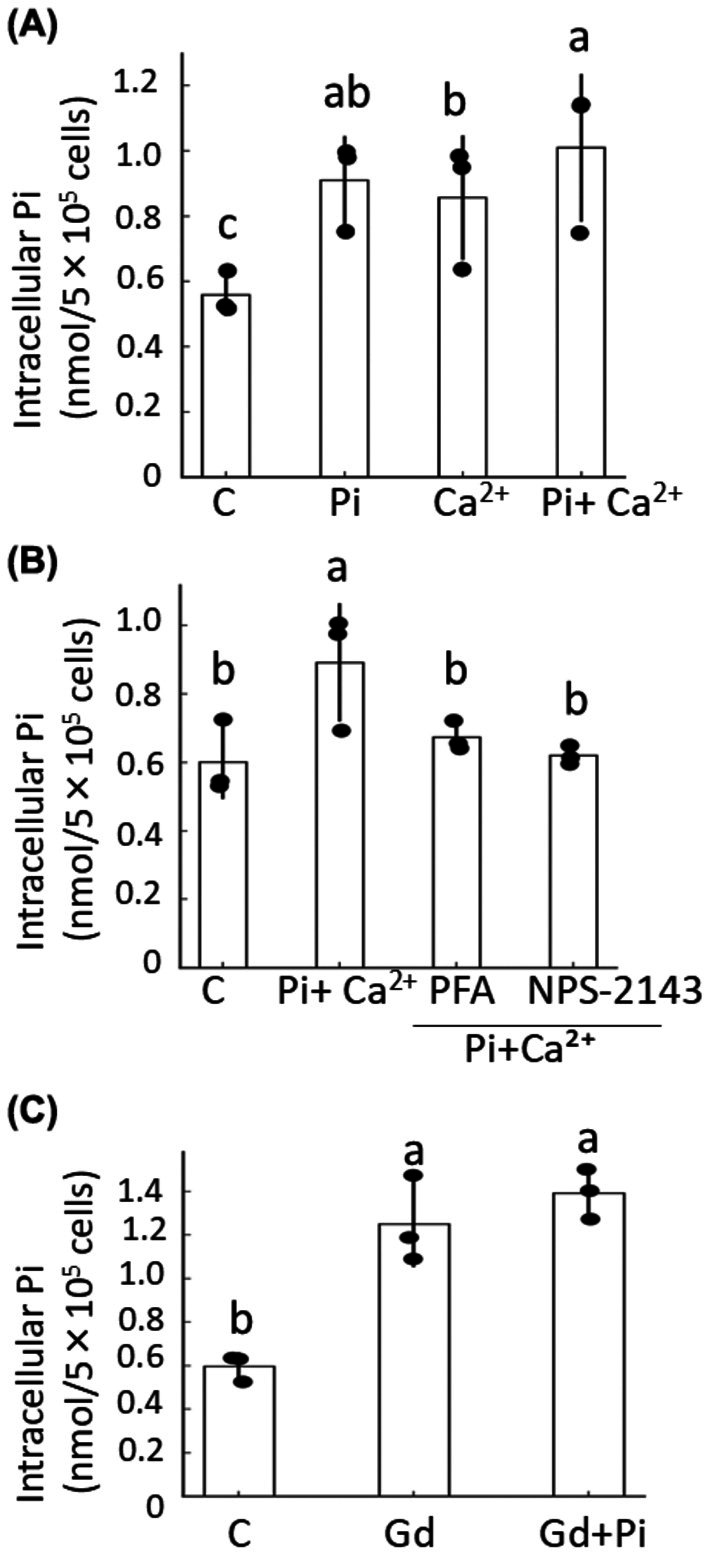
Intracellular Pi concentration in ATDC5 cells. ATDC5 cells were treated for 3 days with 2 mm Pi and 2 mm Ca^2+^, with or without 0.5 mm phosphonoformic acid (PFA, a Pit1 blocker), 0.05 μm NPS2143 (a CaSR antagonist), or 10 μm GdCl₃ (a CaSR agonist), and intracellular Pi concentration was determined using a malachite green assay. (A) Effect of Pi and Ca^2+^ treatment. (B) Effect of PFA and NPS2143 on Pi+Ca^2+^treated cells. (C) Effect of GdCl_3_. Data are shown as mean ± SD (*n* = 3 wells per group), and experiments were repeated three times with similar results. Statistical analysis was performed as described above. Groups labeled with different superscript letters (a, b, c) differ significantly from each other at *P* < 0.05, while groups sharing the same letter do not differ significantly.

Since the intracellular Pi uptake via Pit‐1 causes apoptosis of chondrocytes, we evaluated the intracellular Pi concentration under various conditions. The individual addition of Pi and Ca^2+^ tended to increase the intracellular Pi concentration, and the combined addition of the minerals significantly increased this concentration (Fig. 4A). PFA and NPS‐2143 completely inhibited the minerals' increase of intracellular Pi concentration induced by the combined addition (Fig. 4B). GdCl_3_, an agonist of CaSR [18, 19], enhanced the intracellular Pi concentration (Fig. 4C).

## Discussion

We found that neither Pi nor Ca^2+^ alone caused apoptosis or mineralization; however, their combined presence significantly induced both. Pharmacological inhibition of Pit1 with PFA or CaSR with NPS2143 each abolished the increase in intracellular Pi concentration and apoptosis triggered by Pi + Ca^2+^, whereas activation of CaSR with GdCl_3_ markedly enhanced intracellular Pi accumulation. These results strongly suggest that extracellular Ca^2+^ activates CaSR, which in turn facilitates Pi uptake via Pit1, leading to intracellular phosphate overload and subsequent apoptosis.

Our results support a functional crosstalk between CaSR and phosphate transporters in chondrocytes. While Pit1 has been established as a key phosphate transporter involved in phosphate‐induced stress and apoptosis [4, 14, 16], this study suggests that CaSR activation enhances intracellular Pi uptake through Pit1, representing a previously unrecognized regulatory mechanism of phosphate toxicity in chondrocytes. Crosstalk between phosphate signaling and CaSR has been reported in vascular smooth muscle cells and osteoblasts, where CaSR influences matrix mineralization and cell fate [10, 11, 20]. Our findings extend these observations to chondrocytes, highlighting that CaSR‐Pit1 interactions may be an important determinant of mineral imbalance‐induced cartilage degeneration.

Recent studies have further shown that CaSR is widely expressed in cartilage and bone, modulating chondrocyte differentiation, matrix remodeling, and mechanosensing [12, 13, 21]. High extracellular phosphate, as observed in chronic kidney disease and osteoarthritic cartilage, can alter CaSR signaling and promote apoptosis and pathological calcification [17]. In our model, mineralization was enhanced by Pi + Ca^2+^ cotreatment without a concomitant change in alkaline phosphatase (ALP) activity, a canonical marker of chondrogenic differentiation. This suggests that apoptosis, rather than differentiation, is a primary driver of calcification under these conditions, consistent with emerging evidence that cell death acts as a nucleation event for calcium phosphate deposition [19].

Although we did not directly assess whether combined inhibition of Pit1 and CaSR produces synergistic protection, our findings that blocking either pathway individually abolishes Pi + Ca^2+^‐induced apoptosis strongly support a cooperative mechanism. Future studies using titrated concentrations of PFA and NPS2143 in combination will be required to determine whether these pathways act additively or synergistically. Finally, although we used GdCl_3_ as a CaSR agonist, this compound is not CaSR‐specific, and future experiments employing selective positive allosteric CaSR modulators such as NPSR568 [22, 23] will further strengthen the mechanistic conclusions.

## Conclusions

In conclusion, our findings demonstrate that the combined presence of excess extracellular Pi and Ca^2+^ induces apoptosis and subsequent mineralization in chondrocytes through a cooperative mechanism involving both Pit1 and the Ca^2+^‐sensing receptor (CaSR). Pharmacological inhibition of either Pit1 or CaSR abolished Pi + Ca^2+^‐induced apoptosis, whereas CaSR activation enhanced intracellular phosphate uptake, highlighting functional crosstalk between these pathways. These results provide novel insight into the molecular mechanisms of mineral‐induced cartilage damage and identify CaSR and Pit1 as potential therapeutic targets for the prevention and treatment of pathological cartilage calcification.

## Conflict of interest

The authors declare no conflict of interest.

## Author contributions

Conceptualization, S.N., H.M., M.W., and K.K.; methodology, S.N. and K.K.; validation, H.U., A.K., Y.S., K.I., and S.N.; writing – original draft preparation, H.U. and S.N.; writing – review and editing, K.K.; visualization, H.U. and S.N.; project administration, K.K.; funding acquisition, M.W.

## Data Availability

Data will be made available upon request.
